# In “Cryptic” Population Dynamics, Absence of Evidence Is Not Evidence of Absence

**DOI:** 10.1371/journal.pbio.0050255

**Published:** 2007-09-04

**Authors:** Richard Robinson

When the populations of two species oscillate together, it’s a good bet that they are tightly coupled ecologically. Most often, these oscillations occur between predator and prey (or a pathogen and its host), as demonstrated by the famous case of the Canadian lynx and snowshoe hare, documented in the trapping records of the Hudson’s Bay Company. This makes sense—as the predator depletes the prey, its own reproduction suffers and its numbers decline; as predation eases, the prey’s numbers can grow again.

But is the opposite also true? If the prey’s population doesn’t fluctuate while the predator’s does, may we assume they are not tightly linked in the food web? Among ecologists, this has generally been thought to be the case. But a new study by Takehito Yoshida and colleagues shows that this assumption is not always justified.

The authors noticed some odd population dynamics in studying a rotifer and its food source, a unicellular alga. They observed the pair in a chemostat, an aqueous growth chamber whose nutrient supply is kept steady through a continuous in- and outflow of medium. Observations of the two species have shown that indeed they are tightly linked—the algae are the only food source for the rotifers—and under many conditions within this system, their populations co-oscillate like the lynx and hare. But the authors found that under other conditions, the rotifer’s population waxed and waned, while the alga’s population remained virtually constant.

It seemed plausible that this unexpected result was due to changes in the relative abundances of at least two algal genotypes: one type more palatable to the rotifer predator and one less so. The authors reasoned that when rotifers became abundant, loss of the more palatable prey genotype might be compensated for by growth of the less palatable type, which no longer had to compete for access to nutrients. The numbers of the unpalatable algal type might grow large and, since it was unpalatable, cause the rotifer population to decline, giving advantage again to the more competitive but also more palatable genotype. As a test, they constructed a mathematical model that included terms for prey abundance, palatability, nutrient competition ability, birth rates, and other variables in the system, and found that it predicted that the two algal genotypes could exhibit exactly the observed population behavior when the fitness cost to the algae of being unpalatable was low. Furthermore, the model predicted a similar result for sexually reproducing species, not just asexual ones such as the alga. Low cost of defense against consumption is seen not only in the algae studied by Yoshida and colleagues, but is a widespread observation in many plant–herbivore interactions.

**Figure:**
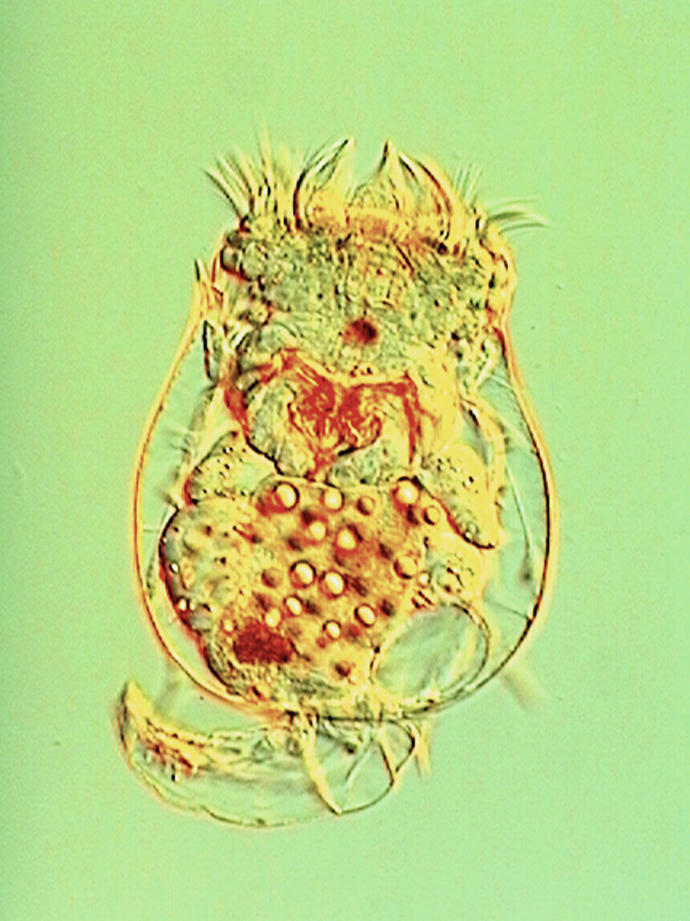
Cryptic cycling: the rotifer *Brachionus* exhibits regular cycles in abundance, but the alga *Chlorella*, on which it feeds, does not—except in its genotype frequencies. The same occurs between the phage T4 and bacteria. (Photomicrograph by T. Yoshida and R. Wayne)

While results from the model were encouraging, the authors did not have direct genotype data from the alga to back it up. Instead, they turned to another pair of interacting species, the bacterium *Escherichia coli* and the phage virus T4. These experiments used two strains of *E. coli*, one of which was resistant to attack by the phage and also carried an easily measured genetic marker. Again, they observed a relatively constant total bacterial population in the face of wide swings in phage abundance and confirmed that the population of the vulnerable bacterial strain showed a typical predator–prey cycle with the phage. In one experiment with only a susceptible strain but in which a spontaneous mutation arose to confer resistance, the total bacterial population ceased to oscillate and again stabilized.

The authors call this pattern of predator–prey population changes “cryptic dynamics,” because the rapid change in relative genotype frequency in the prey masks the strong interaction between the two species. They predict that such a pattern may occur when the prey species is genetically diverse (as is true almost everywhere outside the laboratory), when the better defended (less palatable) genotype is also a poorer competitor for nutrients, and when the cost of defense is low. The implications of this prediction for understanding natural communities are extensive, the authors believe, since the conditions they propose may occur in a large fraction of trophic interactions. In such cases, they argue, lack of population co-oscillations cannot be taken as evidence that trophic interactions are weak: in other words, when it comes to population dynamics, absence of evidence is not evidence of absence.

